# The Role of Pre-Hospital Telecardiology in Reducing the Coronary Reperfusion Time; a Brief Report 

**Published:** 2019-02-03

**Authors:** Peyman Saberian, Nader Tavakoli, Tayeb Ramim, Parisa Hasani-Sharamin, Elham Shams, Alireza Baratloo

**Affiliations:** 1 Prehospital Emergency Research Center, Tehran University of Medical Sciences, Tehran, Iran.; 2 Anesthesiology Department, Imam Khomeini Hospital Complex, Tehran University of Medical Sciences, Tehran, Iran.; 3 Trauma and Injury Research Center, Iran University of Medical Sciences, Tehran, Iran.; 4 Cancer Pharmacogenetics Research Group (CPGRG), Iran University of Medical Sciences, Tehran, Iran.; 5 Tehran Emergency Medical Service Center, Tehran, Iran.; 6 Department of Emergency Medicine, Sina Hospital, Tehran University of Medical Sciences, Tehran, Iran.

**Keywords:** Electrocardiography, Emergency Medical Service, ST Elevation Myocardial Infarction, Telemedicine, Percutaneous Coronary Intervention

## Abstract

**Introduction:**

Telecardiology is defined as using telecommunication for remote treatment of cardiac patients. This study aimed to assess the role of pre-hospital triage via telecardiology on coronary reperfusion time of patients with ST segment elevation myocardial infarction (STEMI).

**Methods:**

This cross-sectional study was conducted from September, 2015 to January, 2018 in six academic referral hospitals, Tehran, Iran. Studied patients were divided into two groups of percutaneous coronary intervention (PCI) following telecardiology or PCI following emergency department (ED) diagnosis of STEMI and time to reperfusion was compared between them.

**Results:**

1205 patients with the mean age of 58.99 ± 12.33 (19-95) years entered the study (82.7% male). 841 (69.8%) cases were transferred directly to the Cath-Lab following telecardiology and 364 (30.2%) cases were first admitted to the ED. There was no significant difference between the groups regarding mean age (p = 0.082) and gender (p = 0.882) of participants. Symptom-to-device interval time in patients who underwent PCI following telecardiology was significantly lower (p < 0.001); however, the difference was not significant in the first medical contact (FMC)-to-device interval time (p = 0.268).

**Conclusions:**

It is likely that the use of telecardiology in pre-hospital triage plays an important role in reducing time to PCI for patients with STEMI.

## Introduction:

Percutaneous coronary intervention (PCI) is recommended as the standard treatment for acute myocardial infarction (MI) (1-3). The time interval from symptom onset until conducting reperfusion is important in reducing the cardiac necrotic area and improving prognosis. Pre-hospital phase is crucial for reducing the time interval between Emergency Medical Service (EMS) contact and cardiac reperfusion. So, it seems that EMS can play an important role in this regard (4). Two important aspects in this phase are the diagnosis of ST segment elevation myocardial infarction (STEMI) and the time interval for transferring the patients to a PCI-equipped center (5). American Cardiac Association, American College Cardiology and Canadian Cardiovascular Committees recommend the use of 12-lead electrocardiogram (ECG) in EMS (1, 4, 6). This allows rapid diagnosis of STEMI in the pre-hospital phase, leading to quick transfer of patients to PCI-equipped facilities instead of hospitals that do not have the facilities, in terms of reducing mortality rates (7, 8). 

Nowadays, many of the EMS ambulances in developed and developing countries have been equipped with 12-lead ECG to reduce reperfusion time. Several strategies for interpreting ECG have been implemented in different countries (9-11). Some are based on the judgment and paraphrasing of the EMS personnel, some based on ECG interpretation by computer to detect acute myocardial infarction and another way is interpretation of pre-hospital ECG by a consultant cardiologist (telecardiology) (12, 13). 

Since 2016, the plan of “code-247” has been implemented by the Iranian ministry of health and medical education, with the aim of reducing the time interval of PCI. The plan is preparation of angioplasty equipped centers for 7 days a week and 24 hours a day to carry out PCI for STEMI patients in the shortest time possible, preferably less than 90 minutes. Since 2017, EMS ambulances have been equipped with a 12-lead electrocardiogram and the ECG report has been sent to a cardiologist. Therefore, STEMI patients have been directly transferred to the angioplasty ward (Cath-Lab) and undergone primary angioplasty. The current study was designed to assess the effect of pre-hospital triage via telecardiology on reperfusion time in patients with STEMI.

## Methods:

***Study design and settings***

This cross-sectional study was conducted from September, 2015 to January, 2018 in six academic referral hospitals (Rasool-e-Akram, Sina, Imam Khomeini, Firoozgar, Tehran Heart Center, Shahid Rajaee) equipped with 24-hour PCI facilities in Tehran, Iran. Studied patients were divided into two groups of PCI following telecardiology or PCI following emergency department (ED) diagnosis of STEMI and the time to reperfusion was compared between them. The study protocol was approved by ethical committee of Iran University of Medical Sciences (IR.IUMS.REC.1397.956). The investigators did not interfere with patients’ management. All the information were anonymous and kept confidential and only generally used and analyzed.

***Participants***

All patients transferred to the mentioned hospitals by EMS with diagnosis of STEMI undergoing PCI were included. Sampling was done in a consecutive manner and there was no exclusion criterion. Patients were divided into two groups: 1) Patients who underwent PCI following telecardiology; 2): Patients who did not undergo an electrocardiogram in an ambulance and were transferred to the ED. 

In the first group, ECG was done on the patient's bedside and sent to a cardiologist in the EMS center, which is known as telecardiology. Then, if the patient was diagnosed with STEMI, the patient was directly transferred to a PCI-equipped center. These patients were directly admitted to Cath-lab and ED was bypassed.

In the second group, patients were transferred to the ED without undergoing ECG on the prehospital setting. The diagnosis of STEMI was made by an ED physician. After primary emergency medical care and cardiologist’s visit, if STEMI was confirmed, he/she would be referred to angioplasty ward.

***Definitions***

*Symptom-to-Device:* The time interval between the onset of patients’ symptoms to PCI.

*First medical contact (FMC)-to-Device:* The time interval between the first time a technician attends the patient’s bedside to PCI.

***Data Collection***

A two-part checklist was prepared for recording patients’ baseline characteristics and main data. The time records in this study included: 1) onset of symptoms; 2) FMC time; 3) PCI time. Arrival and departure to the triage unit, ECG performance, angiographic ward admission and angioplasty times were recorded digitally and manually by the nurses. Registered pre-hospital emergency times, mission forms and time recorded by global positioning system (GPS) were matched to the time recorded in the hospital system for validation of data. 

***Statistical analysis***

Statistical analyses were performed using the IBM SPSS software package, version 22 (SPSS Inc., Chicago, IL, USA). Values were expressed as frequency (number and percentage) or mean ± standard deviation (SD), as appropriate. Chi-square tests were used for comparisons of categorical variables, whereas Mann–Whitney U and its parametric equivalent (Independent T-test) were used to compare numerical variables. Shapiro–Wilks test and Q-Q plot were used to check the normality of the variables; then according to the establishment of assumptions, parametric or nonparametric test was applied. P-value<0.05 was considered statistically significant. 

## Results:

**Figure 1:**
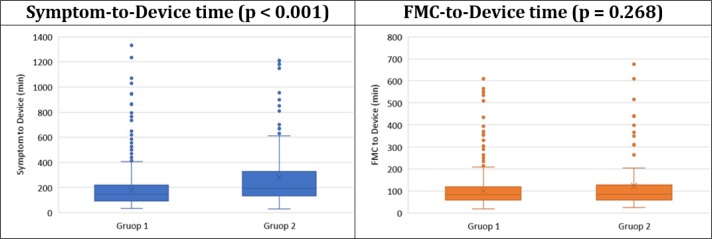
Distribution of symptom-to-Device and first medical contact (FMC)-to-Device times in the two studied groups

A total of 1205 patients with the mean age of 58.99 ± 12.33 (19-95) years entered the study (82.7% male). 841 (69.8%) cases were transferred directly to the Cath-Lab following telecardiology and 364 (30.2%) cases were first admitted to ED. There was no significant difference between groups regarding the mean age (p = 0.082) and gender (p = 0.882) of participants (Table 1). The findings revealed that the incidence of symptoms was more frequent on about 10 am, 4 pm, and 12 pm. Symptom-to-Device time was significantly lower in patients who underwent PCI following telecardiology (p < 0.001); however, the difference was not significant regarding FMC-to-Device time (p = 0.268) (table 1, figure 1). 

## Discussion:

The present study showed that telecardiology in the pre-hospital setting and rapid transportation to the catheterization ward caused a significant reduction in the time to cardiac reperfusion for patients. 

**Table 1:** Demographic characteristics of participants in the two groups

**Variables**	**Telecardiology** ** (n=841)**	**Normal** * ** (n=364)**	**P-value**
**Age (year) **			
Mean ± SD	58.97 ± 12.23	59.04 ± 11.56	0.082
**Gender** **n (%)**			
Male	696 (82.8)	300 (82.4)	0.886
Female	145 (17.2)	64 (17.6)	
**Onset of symptoms (o’clock)**			
0 - 6	204 (24.4)	57 (33.5)	0.006
6 - 12	225 (26.9)	54 (31.8)	
12 - 18	262 (31.3)	34 (20.0)	
18 - 24	145 (17.3)	25 (14.7)	
**Times (minutes)**			
Symptom-to-Device	181.2 ± 139.5	281.6 ± 227.3	<0.001*
FMC-to-Device	100.4 ± 68.7	122.8 ± 113.0	0.268

* Patients who had not undergone electrocardiography in the ambulance and were transferred to the emergency department. FMC: First medical contact. Data are presented as mean ± standard deviation (SD) or frequency (%).

Using telemedicine has been advocated for various fields in existing literature and it has been more than a decade since the first time telemedicine was used to diagnose and treat acute MI (14, 15).

Following a retrospective analysis on 280 consecutive STEMI patients treated with PCI in a rural area of USA, Kahlon et al. found that pre-hospital ECGs decreased FMC-to-Device by 50%, compared with those who were taken to the nearest hospital which was not a PCI-equipped center by the EMS. In our study, although the difference between the two studied groups was significant, it was not 50%. This difference might be due to the fact that all the patients in our study, whether using telecardiology or not, were transferred to a PCI-equipped center, while the studied patients in Kahlon et al. study who did not undergo an ECG in prehospital setting were transferred to a non PCI-equipped center and needed to be transported to another center. In contrast to the Kahlon et al. study in which most of the studied patients (63.4%) had not undergone a prehospital ECG, most of our studied patients (69.8%) had undergone ECG and were transported after using telecardiology (5).

Brunetti et al. conducted a similar study in Italy on 297 consecutive patients with STEMI transferred by EMS and reported that mean time-to-balloon in STEMI patients in ED without tele-medicine support was almost thrice those admitted with pre-hospital triage using telecardiology in the same center. An interesting point in Brunetti et al. study is that both groups were treated within the recommended 90-120 minutes. Unfortunately, in the current survey the mean of Symptom-to-Device intervals in both studied groups were more than 3 hours. This lost time is mostly due to the delay from the onset of the patient's symptoms to the emergency call. However, the FMC-to-Device was almost 2 hours in both groups of the current study, which is considerable and may have resulted from limited PCI-equipped centers, the long distance that the ambulance should usually go from the scene to hospital, and heavy traffic jam in Tehran city (16).

According to previous studies, it's almost safe to say that the pre-hospital triage based on 12-lead electrocardiogram and telecardiology and direct transportation of STEMI patients to hospitals equipped with 24-hour PCI facilities can significantly reduce Symptom-to-Device interval and improve the prognosis of patients with acute MI (17, 18). Also, the findings of the current study showed that equipping ambulances with electrocardiogram device and the possibility of telecardiology by pre-hospital staff reduced the time of the FMC-to-Device. Patients transferred via EMS with telecardiology possibility had a greater chance of cardiac reperfusion in the standard golden time (less than 90 minutes).

It should be noted that the prognosis of patients with acute myocardial infarction is one of the important factors determining the effectiveness of pre-hospital triage design based on electrocardiogram and telecardiology. It is suggested that future studies should aim at assessing the effect of telecardiology on mortality and morbidity rates and overall survival of STEMI patients.

***Limitations***

The process of registration of data in the information system was done by hospital staff so there was no possibility of detecting a registration error. The transfer distance of patients could not be compared due to the lack of transit distance records. In pre-hospital triage based on telecardiology program of EMS of Tehran, patients diagnosed with STEMI were transferred to the PCI-equipped hospitals, which may have been located far away from the scene, instead of non PCI-equipped centers close to the scene. Yet, according to the emergency protocols, patients without telecardiology should be transferred to the closest hospital, which may not have been a 24 hour PCI-equipped center. This can cause bias in comparing FMC-to-Device interval time between our two studied groups. To better compare the effectiveness of telecardiology on FMC-to-Device interval time, it is suggested to compare cardiac patients without telecardiology transferred to non-PCI centers with cardiac patients with telecardiology and transferred to PCI-equipped centers.

## Conclusion:

It is likely that the use of telecardiology in pre-hospital triage plays an important role in reducing time of PCI for patients with acute myocardial infarction with ST segment elevation. Therefore, telecardiology can be used for the suspected STEMI patients to rapidly treat them and reduce complications due to delayed treatment.
